# Beetroot juice — a suitable post-marathon metabolic recovery supplement?

**DOI:** 10.1186/s12970-021-00468-8

**Published:** 2021-12-03

**Authors:** Zinandré Stander, Laneke Luies, Mari van Reenen, Glyn Howatson, Karen M. Keane, Tom Clifford, Emma J. Stevenson, Du Toit Loots

**Affiliations:** 1 Human Metabolomics, North-West University, Potchefstroom, 2531 South Africa; 2 North-West University, Potchefstroom Campus, Private Bag X6001, Box 269, Potchefstroom, 2520 South Africa; 3 Faculty of Health and Life Sciences, Department of Sport, Exercise and Rehabilitation, Northumbria University, Newcastle upon Tyne, NE1 8ST UK; 4 Water Research Group, School of Environmental Sciences and Development, North-West University, Potchefstroom, 2531 South Africa; 5 School of Science and computing, Department of Sport Exercise and Nutrition, Galway Mayo Institute of Technology, Galway, Republic of Ireland; 6 Human Nutrition Research Centre, Faculty of Medicine, Newcastle University, Newcastle upon Tyne, England; 7 School of Sport, Exercise and Health Sciences, Loughborough University, Loughborough, UK

**Keywords:** *Beta vulgaris L.*, Beetroot juice, Endurance running, Recovery, Metabolism, Functional foods

## Abstract

**Background:**

Red beetroot (*Beta vulgaris L.*) is a multifunctional functional food that reportedly exhibits potent anti-inflammatory, antioxidant, vasodilation, and cellular regulatory properties. This vegetable has gained a fair amount of scientific attention as a possible cost-effective supplement to enhance performance and expedite recovery after physical exercise. To date, no study has investigated the effects of incremental beetroot juice ingestion on the metabolic recovery of athletes after an endurance race. Considering this, as well as the beneficial glucose and insulin regulatory roles of beetroot, this study investigated the effects of beetroot juice supplementation on the metabolic recovery trend of athletes within 48 h after completing a marathon.

**Methods:**

By employing an untargeted two-dimensional gas chromatography time-of-flight mass spectrometry approach, serum samples (collected pre-, post-, 24 h post-, and 48 h post-marathon) of 31 marathon athletes that ingested a series (*n* = 7; 250 ml) of either beetroot juice (*n* = 15 athletes) or isocaloric placebo (*n* = 16 athletes) supplements within 48 h post-marathon, were analysed and statistically compared.

**Results:**

The metabolic profiles of the beetroot-ingesting cohort recovered to a pre-marathon-related state within 48 h post-marathon, mimicking the metabolic recovery trend observed in the placebo cohort. Since random inter-individual variation was observed immediately post-marathon, only metabolites with large practical significance (*p*-value ≤0.05 and *d*-value ≥0.5) within 24 h and 48 h post-marathon were considered representative of the effects of beetroot juice on metabolic recovery. These (*n* = 4) mainly included carbohydrates (arabitol and xylose) and odd-chain fatty acids (nonanoate and undecanoate). The majority of these were attributed to beetroot content and possible microbial fermentation thereof.

**Conclusion:**

Apart from the global metabolic recovery trends of the two opposing cohorts, it appears that beetroot ingestion did not expedite metabolic recovery in athletes within 48 h post-marathon.

**Supplementary Information:**

The online version contains supplementary material available at 10.1186/s12970-021-00468-8.

## Background

In addition to the well-established physiological [1] and immunological [2] health effects of endurance running races (> 5 km), this sport induces a wide variety of metabolic adaptations [3] (The acute systematic biochemical adaptations induced by endurance running, submitted). These adaptations are primarily associated with fuel substrate catabolism and typically include elevated glycolysis, lipolysis, amino acid oxidation, ketogenesis, and tricarboxylate cycle (TCA) intermediates [4–8]. Additionally, previously reported elevations in dicarboxylic acids, α-hydroxy acids and odd chain fatty acids coupled with reduced phospholipids, suggest the activation of alternative energy-producing pathways such as ω and/or α-oxidation of fatty acids, as well as autophagy of cellular membranes [4, 9]. These adaptations, along with fluctuations in purine and pyrimidine, urea cycle, and reactive oxygen intermediates [4, 5, 7, 8, 10] not only further attest to the extensive energy-taxing nature of endurance running, but also emphasize the metabolic flexibility of athletes to use multiple fuel substrates during endurance races, to supply the necessary energy to complete the event. Based on previous literature, these metabolic adaptations recover to pre-exercise-related levels within 3–72 h after cessation of endurance exercise [11–14], depending on the intensity, duration, and distance of the running event. Post-exercise metabolic restoration is mainly attributed to a reduction in energy requirements, which leads to the inactivation of catabolic pathways required for energy production, and the activation of anabolic pathways required for recovery, including the nucleotide salvage pathways, glycogenesis, lipogenesis and protein synthesis [11, 12, 14].

Besides the potentially detrimental effects induced by marathons and/or ultramarathons, these are still considered a popular leisure activity, globally. As such, a growing body of research is aimed at identifying more cost-effective strategies that may expedite the recovery process, and subsequently enhancing athlete performance. One of these strategies includes pre- and/or post-exercise ingestion of supplements that either consists of or contains functional foods. Functional foods are primarily defined as any natural and/or fortified foods, rich in bioactive compounds that possess an added advantage to human health besides its nutritional value [15, 16]. Besides the various beneficial health attributes [17–19], phytonutrient-rich fruits and vegetables are also well-known for their radical scavenging, antioxidant, anti-inflammatory and anti-viral/microbial properties [18, 20] that may aid in performance-enhancing and/or physiological recovery of athletes during and/or after exercise. To date, some of the foods that have been investigated for this particular purpose include cherries, blueberries, carrots, bananas, beetroot, blackcurrants, pomegranates, pears, etc. [21–27]. Moreover, it has been proposed that the anti-oxidant and anti-inflammatory capacity of beetroot (*Beta vulgaris L*.) surpasses that of most other fruits and vegetables due to its abundant betalain, among other phytochemicals (polyphenols, vitamin C, rutin, epicatechin, etc.) content, which inhibits cyclooxygenase activity and disrupts nuclear factor kappa-light-chain-enhancer of activated B cells (NF-κB) [19]. In addition to these pigment imposing betalains, beetroot is also considered a Class A performance-enhancing supplement (supported by adequate scientific research) by the Australian Institute of Sport [28] due to its nitrate donating abilities. Nitrate reportedly enhances nitric oxide bioavailability via the nitrate-nitrite-nitric-oxide pathway, subsequently enhancing vasodilation, cellular respiration regulation and neurotransmission [21, 29, 30]. Besides the successful application of beetroot juice as a performance-enhancing supplement, controversial evidence exists regarding its ability to expedite the physiological recovery process after exercise [21, 31]. To our knowledge, no study has investigated the capacity of beetroot juice to facilitate metabolic recovery after endurance running.

Thus, in this double-blinded, placebo-controlled investigation, an untargeted two-dimensional gas chromatography time-of-flight mass spectrometry approach (GCxGC-TOFMS) metabolomics approach was used to determine whether or not beetroot juice ingestion possesses an added advantage towards metabolic recovery when compared to unaided recovery within 48 h post-marathon. This knowledge could not only further improve the current understanding of beetroot aided recovery, but also provide clues to new, more effective beetroot supplementation strategies after exercise.

## Methods and materials

Considering that this project forms part of a larger study that consists of multiple (inter-disciplinary) aims, participant recruitment and selection information [21], additional clinical and physiological measurements [21], as well as complementary metabolic investigations [4, 14] based on sub-divisions of the current cohort, have already been published and may be referred to for additional information not pertinent to this investigation.

### Participants

The participants included in this investigation were selected at random and participation was completely voluntary. Prior to the marathon, all participants were required to complete a health and dietary questionnaire (with an additional menstrual cycle questionnaire for female participants), of which individuals receiving or using any anti-inflammatory treatments, chronic medication, as well as those with any food allergies, cardiovascular complications or musculoskeletal disorders and injuries were excluded from the study. All athletes were instructed to refrain from exercising and/or using any alternative recovery modalities (heat, cryotherapy, inflammatory drugs, antioxidant vitamins, compression garments, etc.) during the recovery period of this investigation. Withal, the use of anti-bacterial mouth wash was prohibited as a means of conserving the proposed bacterial nitrate-nitrite conversion of beetroot juice in the oral cavity. All the participants gave written and informed consent before the commencement of any analysis. An overview of the participant characteristics/demographics is presented in Table 1.

**Table 1:** A summary of the participant characteristics of the placebo and beetroot ingesting cohorts

Participant demographical information	Placebo cohort *(n = 16)*	Beetroot cohort *(n = 15)*
	Average ± standard deviation	
Age (years)	39 ± 12	42 ± 10
Pre-marathon athlete weight (kg)	72.2 ± 11.9	70.3 ± 7.9
Post-marathon athlete weight (kg)	70.8 ± 11.7	69 ± 7.4
Marathon experience (years)	8 ± 7	11 ± 10
Finishing time (hh:mm:ss)	04:30:25 ± 00:36:48	04:07:08 ± 00:39:16

### Clinical samples and supplementation

Blood samples of 31 athletes (19 males; 12 females) were obtained (antecubital venesection) before (P0), after (P1), as well as 24 h (P2) and 48 h (P3) after completing the Druridge Bay Marathon (Northumberland, UK) [21]. During the two consecutive days following the race, athletes received either beetroot juice (*n* = 15 athletes; 9 males and 6 females) or isocaloric placebo supplements (*n* = 16 athletes; 10 males and 6 females). Placebo samples consisted of a maltodextrin, protein powder, and fruit squash mixture, with a similar macro-nutrient content to that of the beetroot juice supplement (containing approximately 400 mg of phenolic compounds and 194 mg of the pigment, betanin), as described by Clifford, Allerton [21] and indicated in Table S2. These supplements were placed in containers that were indistinguishable in appearance and were consumed as follows: 3 × 250 ml supplements on the day of the marathon (immediately after P1 sampling, ±3 h post-race, and at 20:00), 3 × 250 ml supplements on the first day after the marathon (upon waking-up, with lunch, and with supper), and 1 × 250 ml supplement upon waking on the second day post-marathon. Participant groups were matched according to predicted marathon finishing times and did not significantly differ in terms of recorded dietary intake (determined using Nutritics dietary analysis software), or the number of males/females per group [21]. P0 samples were collected at participant-convenient times preceding the race and patients were required to be in a hydrated yet fasted (for at least 4 h) state, whereas P1 samples were acquired within 30 min after completing the race, thus dictating the approximate time of P2 and P3 collection. All the blood samples were collected in 10 mL vacutainer vials and placed on ice before being transported to the Northumbria University (Newcastle upon Tyne, UK), Faculty of Health and Life Sciences (Department of Sport, Exercise and Rehabilitation) laboratory, for further processing. Initial sample processing included clotting at room temperature for 30 min, followed by a 10 min centrifugation step (3000 *g*)*.* The serum (supernatant) was then extracted, immediately frozen (− 80 °C), and transported on dry ice to the North-West University, Human Metabolomics: Laboratory of Infectious and Acquired Diseases for metabolomics analyses. All samples were stored at − 80 °C until analysis commenced. A schematic representation of the larger metabolic study design is presented in Fig. S1 of this investigation and the Supplementary material of Stander, Luies [14].

### Total metabolome extraction and derivatisation

As previously described [4, 14], all samples, including pooled quality control samples (containing 50 μl of each sample), were subjected to an in-house total metabolome extraction (SOP number: HM-MET-056) and traditional TMCS derivatisation before being analysed. To summarise; 50 μl of internal standard (3-phenylbutyrate; 0.45 μg/ml), dissolved in a chloroform:methanol:milliQ water (1:3:1) solution, was added to smaller aliquots (50 μl) of the samples. While on ice, 300 μl of ice-cold acetonitrile was added to the aliquots, whereupon it was mixed for 2 min (REAX D-91126 vortex; Heidolph Instruments GmbH & Co.KG, Schwabach, Germany), and centrifuged for 10 min at 4000 rpm. The supernatants of the samples were then extracted, transferred to glass GC-MS vials, placed in a heating block set to 40 °C, and dried under a stream of nitrogen gas for approximately 45 min. Using a Hamilton syringe, 25 μl of methoxamine hydrochloride (dissolved in 15 mg/ml pyridine) was added to each vial, which proceeded to incubate for 90 min at 50 °C. Finally, samples were derivatised with 40 μl BSTFA (enriched with 1% TMCS) for 60 min at 60 °C, before being transferred to a new GC vial containing a vial insert.

### GCxGC-TOFMS analysis and processing

The randomised samples were injected (1 μl; 1:3 split ratio) into the Pegasus 4D GCxGC-TOFMS system (LECO Africa (Pty) Ltd., Johannesburg, South Africa), using the Gerstel auto-sampler (Gerstel GmbH and co. KG, Mülheim van der Ruhr, Germany). The carrier gas (purified helium) was set to flow at a constant rate of 1 ml/min, while the injector temperature was held at 270 °C. The primary oven, containing a Restek Rxi-5MS capillary column (30 m; 0.25 μm diameter and 0.25 μm film thickness), was programmed with an initial temperature of 70 °C, which incrementally (4 °C/min) increased until a final temperature of 300 °C was reached (maintained for 2 min). The secondary oven, containing a Restek Rxi-17 capillary column (1 m; 0.25 μm diameter and 0.25 μm film thickness), was set at 85 °C, which increased with 4.5 °C/min until a final temperature of 300 °C was reached (maintained for 2 min), while the thermal modulator pulsed cold and hot streams nitrogen gas every 3 s for a duration of 0.5 s. The mass spectra (ms) of the first 400 s of each run was discarded (regarded as solvent delay), whereafter ms of ions (50–800 m/z) were acquired at 200 ms/s. The transfer line and ion source were held at 270 °C and 220 °C, respectively, with a detector voltage of 1600 V and filament bias of − 70 eV. The data generated from the GCxGC-TOFMS was processed (deconvolution, peak alignment and identification) using the ChromaTOF Software (LECO Corporation), as described by Stander, Luies [4].

### Data processing and statistical analyses

The dataset obtained was normalised relative to the internal standard, and plasticizers, analytical contaminants, and column-related compounds were removed. Hereafter, a 50% zero value filter, zero value replacement (with random values below the detection limit), 50% quality control coefficient of variation (QC-CV) filter (retaining metabolites with a CV ≤ 50%), log transformation, and auto-scaling were performed.

Following these clean-up steps, the data was subjected to a variety of multivariate and univariate statistical methods using MATLAB [32] (in conjunction with a PLS [33] toolbox), as a means of selecting those metabolites pertinent to the aim of this investigation. To comprehensively address the aim of this investigation, multiple statistical objectives, and therefore comparisons, were required. In summary, paired statistical analysis of the beetroot juice-ingesting cohort was performed to confirm whether this cohort indeed recovered to a pre-marathon-related state within 48 h (statistical objective A), as has already been confirmed for the placebo group [14]. Hence, the P2 and P3 serum metabolite profiles of the beetroot-ingesting cohort were respectively compared to that of the P0 profile to identify any differentiating metabolites which would oppose metabolic recovery. Multivariate analyses included multilevel (ML) principal component analyses (PCA) and ML-partial squares discriminant analysis (ML-PLS-DA), while univariate analyses consisted of a paired t-test, and effect size tests, to assess statistical and practical significance respectively. To control for false discovery rates (FDR) associated with large scale multiple testing, as in the case of untargeted metabolomics datasets, t-test *p*-values were adjusted using the Benjamini-Hochberg procedure (limiting FDRs to 5%). All metabolites (P0 vs P2 and P0 vs P3) with a BH adjusted *p*-value ≤0.05 and an effect size *d*-value ≥0.5 were deemed significant.

To determine whether or not beetroot juice ingestion expedites the metabolic recovery trend of athletes within 48 h post-marathon, unpaired statistical analyses of the beetroot cohort vs placebo cohort were performed (statistical objective B). Foremost, the metabolic progression for both treatment groups over time was compared by using statistical models that accounted for the entire experimental design and dependencies between measures, i.e. a two-way repeated-measures analysis of variance (RM ANOVA), and an unfolded PCA. The latter transforms a three-dimensional tensor into a two-dimensional matrix (Fig. S2), thus allowing for PCA [34]. To supplement these comprehensive statistical methods, recovery time-point-specific inter-cohort comparisons were performed to assess day-specific variation that may be apparent between the cohorts. For this, multivariate statistical methods included PCA, PLS-DA, whilst univariate methods comprised an independent samples t-test (FDR’s controlled to 5% according to the BH procedure), and independent effect size tests based on Cohen’s *d*-values. Here, variables were selected based on a BH adjusted *p*-value ≤0.05 or a Cohen’s *d*-value ≥1.0, thus considering both statistical and practical significance. As opposed to the equal statistical and practical relevance of marker selection in objective A, this objective’s selection was more stringent on practical relevance to capture slight differences that may be of practical importance.

Since the cohort sizes of this investigation are relatively small, multivariate models are less readily validated and could therefore only be used to visualise trends and variation, whilst univariate models, better equipped to avoid false discoveries, were employed for variable selection in both statistical objectives.

## Results

**Fig. 1:**
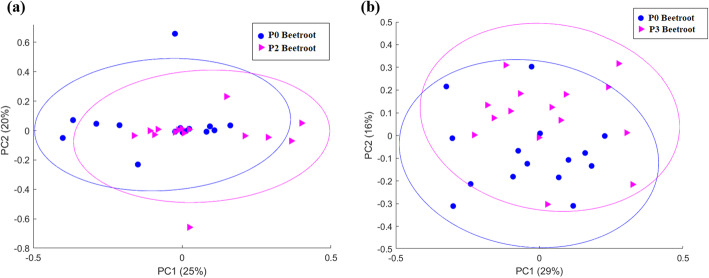
Multi-level principal component analysis plot of the pre-marathon [P0] serum metabolite profiles of the beetroot group relative to the (**a**) 24 h post-marathon [P1] and (**b**) 48 h post-marathon metabolite profiles [P2], profiles of the same group. Abbreviations: PC: principal component. The ellipsoids represent 90% confidence intervals of each centroid

When comparing both the P2 and P3 serum metabolite profiles of the beetroot cohort to the corresponding P0 profiles (statistical objective A), no metabolite markers were considered statistically significant (BH *p*-value ≤0.05 and *d*-value ≥0.5), suggesting that the metabolome of the beetroot cohort recovered to a baseline-related state within 24 h post-marathon. This was further indicated by the confined positioning of the respective ML-PCA of each recovery time-point relative to the P0 ellipsoids, as presented in Fig. 1.

**Fig. 2:**
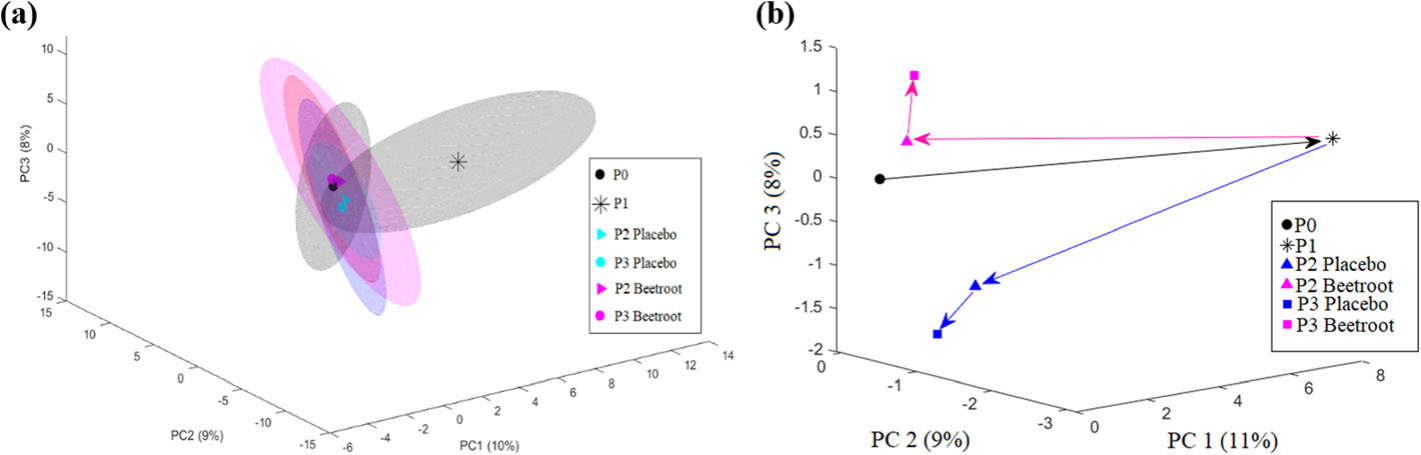
Unfolded principal component analysis (**a**) with, and (**b**) without confidence intervals, depicting the global recovery trend of the beetroot and placebo cohort over time. Abbreviations: P0: pre-marathon; P1: post-marathon; P2: 24 h post-marathon; P3: 48 h post-marathon; PC: principal component. The ellipsoids in (**a**) represent 90% confidence intervals of each centroid

Since these results correspond with the findings of Stander, Luies [14] that indicated overall metabolic restoration of the placebo athletes within 48 h post-marathon, an unfolded PCA was constructed to visualise the time-dependent global metabolic recovery trend of the two cohorts relative to each other (Fig. 2) (statistical objective B).

Based on the unfolded PCA plots it is evident that the most significant effect emanated from the marathon perturbation [4], after which both groups returned to a pre-marathon-related state within ±24 h [14]. Intensely overlapped confidence intervals of the recovery time-points of the beetroot juice and placebo cohorts suggest little to no variation between groups at these times (Fig. 2a). However, upon removal of confidence intervals, P2 and P3 centroids of the beetroot cohort are seemingly slightly closer to that of P0 (Fig. 2b), which could be indicative of small inter-cohort differences. As such, a two-way RM ANOVA was employed, and 16 metabolites were identified as having a statistically significant (*p*-value ≤0.05) interaction (between time and intervention) and/or intervention effect (Table 2). Considering that this univariate method focuses on relatively small differences within the entire study design (P0–P3), the RM ANOVA alone does not discriminate the precise origin of the metabolic fluctuation observed. Consequently, pairwise comparisons of the different time-points were employed to determine day-specific recovery effects. Whilst the PCA plots of each time-point comparison (Fig. S3) did not visually portray significant differences between the groups at the different time-points, Cohen’s *d*-values for the majority of these RM ANOVA metabolites indicated random, moderate-to-large practical variance amongst the groups (Table 2). A more detailed table including RM ANOVA *p*-values as well as average metabolite concentrations and standard deviations can be found in Table S1.

**Table 2:** Metabolites varying significantly between the beetroot and placebo cohorts, according to RM ANOVA and day-specific pairwise comparisons

Metabolite ***(PubChem ID)***	P0 Placebo vs P0 Beetroot juice		P1 Placebo vs P1 Beetroot juice		P2 Placebo vs P2 Beetroot juice		P3 Placebo vs P3 Beetroot juice	
	BH ***p-***value	Cohen’s ***d***-value	BH ***p***-value	Cohen’s ***d***-value	BH ***p***-value	Cohen’s ***d***-value	BH ***p***-value	Cohen’s ***d***-value
α-Oleoylglycerol *(5319879)* ^*b*^	0.89	0.65	0.92	0.31	1.00	0.13	0.89	0.19
β-Hydroxydecenedioate *(121232666)* ^*b*^	0.92	0.33	0.97	0.05	1.00	0.37	0.56	0.44
β-Hydroxyphenylacetate *(12122)* ^*a*^	0.97	0.22	0.92	0.60	1.00	0.34	0.27	0.85
β-Hydroxyvalerate *(107802)* ^*a*^	0.92	0.33	0.92	0.54	1.00	0.58	0.31	0.64
*p*-Hydroxyphenylacetate *(127)* ^*a*^	0.89	0.61	0.92	0.31	1.00	0.65	0.30	0.70
Arabitol *(43925)* ^a,b,*^	0.89	0.45	0.96	0.12	**0.03**	**1.40**	**2.1 × 10**^**−3**^	**1.73**
Eicosanoate *(10467)* ^*a*^	0.89	0.58	0.92	0.46	1.00	0.36	0.50	0.56
Glycerol *(753)* ^*b*^	0.97	0.13	0.92	0.57	1.00	0.24	0.99	0.02
Hippurate *(464)* ^*a*^	0.89	0.59	0.92	0.39	1.00	0.19	0.27	0.86
Mannitol *(6251)* ^*a*^	0.99	0.11	0.92	0.37	1.00	0.55	0.37	0.59
Nonanoate *(8158)* ^a,*^	0.89	0.71	0.92	0.84	0.57	**1.02**	**2.6 × 10**^**−3**^	**1.63**
Oxalate *(971)* ^*a*^	0.89	0.40	0.92	0.79	1.00	0.49	0.92	0.12
Rhamnose *(25310)*	0.89	0.63	0.92	0.46	1.00	0.65	0.88	0.22
Threonate *(21145021)* ^*a,b*^	0.88	0.61	0.89	1.03	1.00	0.30	0.52	0.58
Undecanoate *(8180)* ^a,*^	0.40	1.24	0.92	1.07	1.00	0.35	**0.04**	**1.38**
Xylose *(135191)* ^a,b,*^	0.89	0.56	0.92	0.29	0.21	**1.04**	0.53	0.46

Intervention (^a^), interaction (^b^) effects, and day-specific significance (*) are indicated in the first column, while day-specific *p*- and *d*-value significance are indicated in bold

In light of the pre-existing individual variance (particularly at P1), as well as the lack in statistical significance of metabolites (BH *p*-value ≤0.05) during the recovery period (P2 and P3), only those metabolites with a large effect (*d*-value ≥1.0) at the respective recovery time-points were considered relevant in terms of understanding any possible treatment-induced differences at various stages of recovery (P2 and P3), when comparing the beetroot and the placebo cohort. Based on this, only four metabolites (arabitol, nonanoate, undecanoate and xylose) were identified as significantly altered when comparing these cohorts (indicated with an asterisk in Table 2), while significant *p*- and *d*-values are emphasized in bold.

## Discussion

Beetroot juice has gained a considerable amount of attention as a possible performance optimisation and recovery enhancing sports supplement due to its potent reactive oxygen scavenging properties and its ability to promote vasodilation, regulate cellular respiration and reduce inflammation [19, 35]. However, limited literature is available pertaining to the metabolic recovery effects of beetroot juice after endurance running exercises. In order to completely ascertain whether or not beetroot juice possess an added advantage in terms of metabolic recovery within 48 h post-marathon, it is imperative to also comprehend the unaided metabolic recovery of athletes within the same timeframe. These results have been published as part of a previous investigation [14], and will therefore only be summarised here. Unaided metabolic recovery was attained within 24 h post-marathon (with exception of xylose), and included reductions in carbohydrates, fatty acids, and TCA cycle intermediates, along with elevations in amino acids. Recovery of these was ascribed to a reduction in energy requirements, leading to the activation of glucogenesis, fatty acid re-esterification, protein synthesis, cellular membrane restoration and the possible activation of nucleotide salvage pathways [14, 36]. In this investigation, a comprehensive comparison of the recovery trends of the beetroot juice and placebo cohorts did not vary significantly (Fig. 2), thus indicating no added advantage of beetroot juice ingestion in terms of metabolic recovery after the marathon. However, when employing a two-way RM ANOVA, significant (albeit small) concentration fluctuations were observed in 16 metabolites (Table 2). Whilst these fluctuations were significant when considering the entire study design, pairwise analysis of the respective time-points later revealed that a large portion is owed to P1 (and to some extent P0) variation between athletes. Besides the inevitable confounder of genetic, age and gender variation, these P1 disparities are to be expected since the dietary regimens of athletes were not restricted and/or controlled at either of these time-points as a means of providing a more relatable, robust representation of the marathon perturbation [4]. Regardless, while the substantial impact of the marathon perturbation overshadowed these differences [4], the effect of beetroot juice supplementation is evidently negligible [21, 37]. This is further supported in the study performed by Clifford, Allerton [21], in which beetroot juice supplementation did not attenuate muscle soreness or inflammation, nor did it improve muscle functionality in the same set of athletes. As such, it is unsurprising that only four metabolite markers were identified to vary significantly at the different stages of recovery (P2 and P3). These metabolites (arabitol, xylose, nonanoate, and undecanoate) will be comprehensively discussed below.

In addition to the initial increase of arabitol in both cohorts during the marathon [4], this metabolite continued to increase in the beetroot-ingesting cohort during the recovery period, while contrarily decreasing in the placebo cohort [14]. Although at first glance this may be associated with low-calorie sweetener ingestion, as previously reported to occur during the marathon [4], *Love Beets* product labels indicated that no additional sweeteners were added to this 99% organic beetroot juice supplement (with 1% lemon juice as a stabiliser). However, considering that red beetroot pectin fibres are rich in various sugars including xylose, rhamnose, mannose and especially, arabinose [38], it can be deduced that although not directly added to the supplement, arabitol may have been produced via the reduction of arabinose in the polyol pathway present in humans and various intestinal yeasts [39, 40]. Furthermore, it has been reported that intestinal bacteria preferentially ferment arabinose over other sugars such as xylose [41], thus further ascribing the elevated xylose bioavailability at P2 in this cohort. In addition, xylose can serve as a minor precursor for ribose, the latter which is a key component in the uridine-dependent salvage pathway during recovery [14]. As such, since beetroot phytonutrients, including betaines [42], folate [43], and glutamine [38], can assist in the process of nucleotide restoration, the observed P2 elevations in xylose could also partially be indicative of the role of beetroot-promoting nucleotide synthesis via alternative constituents, subsequently reducing the incorporation of xylose to produce ribose. Although still being significantly elevated compared to the placebo cohort, slight P3 reductions (compared to P2) in both arabitol (significant) and xylose (non-significant) were observed in the beetroot juice cohort and may be attributed to the fact that only a single beetroot supplement (upon waking) was ingested on the final day of the investigation (P3), as opposed to the three servings at P1 and P2. Nevertheless, arabitol has been associated with numerous health benefits, of which the prevention of blood sugar dysregulation and fat deposition in the intestinal tract [40] is the most applicable to post-race metabolic recovery.

Opposing fluctuations in OCFA (nonanoate and undecanoate) concentrations were observed during recovery. In general, reductions in undecenoate (C_11_) observed in both cohorts within 24 h post-marathon, is most likely due to residual catabolism of the accumulated fatty acids via α-oxidation [4]. This is further supported by elevations in nonanoate (C_9_) in both cohorts during this time since it is a downstream product of undecanoate catabolism. Nevertheless, mitigation of OCFA catabolism is evident by the reduction in both nonanoate and undecanoate concentrations in the placebo cohort within 48 h post-marathon. Contrarily, however, both of these OCFAs are elevated in the beetroot cohort 48 h post-marathon, possibly indicating the activation of OCFA synthesis in this cohort. The proposed mechanism of action for this includes the fermentation of beetroot sugar-content to propanoate via intestinal bacteria (*Bacteriodetes, Clostridium, Lactobacillus,* etc.) [44], which can be further utilised for OCFA synthesis. Argumentatively, placebo supplements evidently contained the same total amount of sugar, however, this did not correlate with an increase in OCFAs 48 h post-marathon. This may not only be ascribed to the increased propionate-producing bacterial activity (i.e. *Lactobacillus)* induced by beetroot pectin ingestion [44] but may also arise as a result of the polyphenol content in beetroot which may hinder effective carbohydrate absorption in the intestines [45], subsequently prolonging the exposure of beetroot sugars to gut microbes for fermentation. Besides the possible contribution of OCFA to phospholipid content (i.e. cellular membrane restoration), recent findings have observed an inversed correlation of OCFA and leptin as well as plasminogen-inhibitor-1 [46], further suggesting its role in energy homeostasis regulation and the activation of fibrinolysis. Whether or not elevated OCFAs has an additional advantage pertaining to athlete recovery, remains to be determined.

As with all human-based investigations, an inevitable limitation includes human genotype/phenotype (sex, age, and ethnicity [latter not recorded]) variation confounders, as well as possible dietary variation among athletes. Considering the list of restrictions in terms of recovery modalities and prohibition of anti-bacterial mouth wash provided to the athletes, additional dietary restrictions would have potentially imposed on personalised regimens of these athletes, leading to a reduction in the already confined cohort size. Although not controlled, dietary intake was recorded for the entire duration of this investigation in an attempt to circumvent this. However, these individual variations may introduce a higher level of robustness to the results, providing a more realistic and relatable representation of the metabolic effects of incremental beetroot juice supplementation after a marathon. Since the cohort size of this investigation is considered relatively small in terms of human-based studies and the intricate accompanying statistical approaches needed for analysing the data, future studies consisting of a larger cohort (particularly per gender), could further validate these findings and possibly determine the metabolic effects of beetroot juice in a gender-dependent manner. Additionally, the lack of efficacy of beetroot juice observed in this study may be due to the rapid restoration of the metabolism even when not subjected to aiding strategies as well as restricted bioavailability of certain phytonutrients [47, 48] especially at lower dosage administrations [35]. As such, future studies may consider observing the effect(s) of beetroot supplements at shorter incremental time-points along this recovery process (i.e. 6 h, 12 h, 18 h, etc.) and/or at higher dosages. Furthermore, since the microbiome of athletes seems to be a key component in the conversion of red beetroot nitrate, the fermentation of sugar components, and metabolism of polyphenols, metabolic studies investigating the effects of beetroot on the gut microbiome and microbial function, could determine the fermentation preferences and possible causes for metabolic delays pertinent to phytonutrient uptake.

## Conclusion

This investigation not only supported previous findings of total metabolic recovery of athletes within 48 h post-marathon but further indicated that beetroot ingestion does not expedite the metabolic recovery process. Although several significant, albeit small, metabolic fluctuations between cohorts were selected based on univariate statistics, the majority revealed considerable random variation at P1, which is owed to opposing athletic regimens and inherent biological and physiological aspects. As such, only metabolites with large practical significance at the respective days of the supplement interventions (P2 and P3) were considered when addressing the aim of this investigation. Of the four metabolites identified, the majority were associated with the glycaemic content of red beetroot, as well as the increased microbial activity stimulated by this vegetable. Whether these may provide an additional advantage during an extended period of recovery remains unknown, but as for immediate metabolic recovery, no additional advantage associated with these metabolites were annotated. However, the lack of beetroot juice effectiveness may stem from previously described restricted bioavailability and possible dose-dependent functionality of certain phytonutrients central to the recovery process, and the considerable dependence on the composition and fermentation preferences of gut microbes.

## Supplementary Information

**Additional file 1.**

## Data Availability

As the current investigation is part of a larger collaboration study consisting of multiple aims, the dataset of this investigation is not yet publicly available but can be acquired from the corresponding author on reasonable request. The authors declare that all the results included in this study have been presented clearly, honestly and without fabrication, falsification, or inappropriate data manipulation.

## Abbreviations

BH: Benjamini-Hochberg
BSTFA: N,O-Bis(trimethylsilyl)trifluoroacetamide
CV: Coefficient of variance
DNA: Deoxyribonucleic acid
FDR: False discovery rates
GC: Gas chromatography
GCxGC-TOFMS: Two-dimensional gas chromatography time-of-flight mass spectrometry
ML: Multilevel
MS: Mass spectrometry
OCFA: Odd-chain fatty acids
P0: Pre-marathon
P1: Post-marathon
P2: 24 h post-marathon
P3: 48 h post-marathon
PCA: Principal component analysis
PLS-DA: Partial least squares discriminant analysis
QC: Quality Control
RM ANOVA: Repeated-measures analysis of variance
TCA: Tricarboxylic acid
TMCS: Trimethylchlorosilane
