# Supplementary material for: Beetroot juice — a suitable post-marathon metabolic recovery supplement?
Source: J Int Soc Sports Nutr. 2021 Dec 3;18:72. doi: 10.1186/s12970-021-00468-8 (PMC8642879; doi:10.1186/s12970-021-00468-8)
Supplement: Supplementary file 1 — Additional file 1. [file 12970_2021_468_MOESM1_ESM.docx]

# SUPPLEMENTARY MATERIAL

***Table S1****: Average concentrations and RM ANOVA p-values the metabolites selected to be significant pertaining to the aim of this investigation*

| **Metabolite *(PubChem ID)*** | **Group** | **RM ANOVA** | | **P0** | **P1** | **P2** | **P3** |
| --- | --- | --- | --- | --- | --- | --- | --- |
|  |  | ***Intervention p-value*** | ***Interaction p-value*** | ***Average concentration*** ±***standard deviation*** | | | |
| α-Oleoylglycerol *(5319879)* | *Placebo* | 0.42 | **0.01** | 0.001 ± 3.8×10^-4^ | 0.001 ± 3.6×10^-4^ | 4.3×10^-4^ ± 3.1×10^-4^ | 0.001 ± 3.9×10^-4^ |
|  | *Beetroot* |  |  | 0.001 ± 0.001 | 4.1×10^-4^ ± 2.6×10^-4^ | 4.8×10^-4^ ± 3.2×10^-4^ | 0.001 ± 3.9×10^-4^ |
| β-Hydroxydecenedioate *(121232666)* | *Placebo* | 0.58 | **0.04** | 0.005 ± 0.001 | 0.006 ± 0.002 | 0.007 ± 0.002 | 0.005 ± 0.001 |
|  | *Beetroot* |  |  | 0.006 ± 0.002 | 0.006 ± 0.002 | 0.006 ± 0.001 | 0.006 ± 0.003 |
| β-Hydroxyphenylacetate *(12122) ^a^* | *Placebo* | **0.01** | 0.07 | 3.1×10^-5^ ± 4.6×10^-5^ | 2.8×10^-5^ ± 4.1×10^-5^ | 1.6×10^-5^ ± 3.1×10^-5^ | 2.0×10^-5^ ± 3.6×10^-5^ |
|  | *Beetroot* |  |  | 4.3×10^-5^ ± 6.4×10^-5^ | 1.1×10^-4^ ± 1.3×10^-4^ | 3.2×10^-5^ ± 4.3×10^-5^ | 5.9×10^-5^ ± 4.3×10^-5^ |
| β-Hydroxyvalerate *(107802)* | *Placebo* | **0.02** | 0.72 | 5.3×10^-4^ ± 4.2×10^-4^ | 6.0×10^-4^ ± 3.9×10^-4^ | 6.8×10^-4^ ± 4.5×10^-4^ | 5.2×10^-4^ ± 1.7×10^-4^ |
|  | *Beetroot* |  |  | 0.001 ± 0.001 | 0.002 ± 0.002 | 0.001 ± 0.001 | 0.001 ± 0.001 |
| *p*-Hydroxyphenylacetate *(127)* | *Placebo* | **0.02** | 0.92 | 0.001 ± 0.001 | 0.002 ± 0.002 | 0.001 ± 0.001 | 0.001 ± 0.001 |
|  | *Beetroot* |  |  | 0.002 ± 0.001 | 0.003 ± 0.001 | 0.002 ± 0.001 | 0.002 ± 0.001 |
| Arabitol *(43925)* | *Placebo* | **2.9×10^-4^** | **1.4×10^-8^** | 0.004 ± 0.001 | 0.005 ± 0.002 | 0.004 ± 0.001 | 0.003 ± 0.001 |
|  | *Beetroot* |  |  | 0.005 ± 0.002 | 0.005 ± 0.002 | 0.007 ± 0.002 | 0.007 ± 0.002 |
| Eicosanoate *(10467)* | *Placebo* | **0.01** | 0.95 | 4.2×10^-4^ ± 2.3×10^-4^ | 0.001 ± 4.1×10^-4^ | 4.6×10^-4^ ± 2.9×10^-4^ | 3.7×10^-4^ ± 2.0×10^-4^ |
|  | *Beetroot* |  |  | 0.001 ± 4.4×10^-4^ | 0.001 ± 3.6×10^-4^ | 0.001 ± 0.001 | 0.001 ± 2.8×10^-4^ |
| Glycerol *(753)* | *Placebo* | 0.33 | **0.03** | 0.084 ± 0.031 | 0.492 ± 0.157 | 0.083 ± 0.032 | 0.092 ± 0.036 |
|  | *Beetroot* |  |  | 0.092 ± 0.052 | 0.399 ± 0.114 | 0.091 ± 0.032 | 0.094 ± 0.047 |
| Hippurate *(464)* | *Placebo* | **0.02** | 0.73 | 0.002 ± 0.002 | 0.003 ± 0.004 | 0.003 ± 0.004 | 0.001 ± 0.002 |
|  | *Beetroot* |  |  | 0.003 ± 0.003 | 0.005 ± 0.006 | 0.004 ± 0.004 | 0.004 ± 0.003 |
| Mannitol *(6251)* | *Placebo* | **0.01** | 0.06 | 0.003 ± 0.004 | 0.005 ± 0.004 | 0.004 ± 0.003 | 0.003 ± 0.003 |
|  | *Beetroot* |  |  | 0.004 ± 0.004 | 0.01 ± 0.013 | 0.02 ± 0.03 | 0.008 ± 0.009 |
| Nonanoate *(8158)* | *Placebo* | **1.7×10^-3^** | 0.13 | 0.004 ± 0.002 | 0.004 ± 0.002 | 0.004 ± 0.001 | 0.003 ± 0.001 |
|  | *Beetroot* |  |  | 0.005 ± 0.002 | 0.005 ± 0.001 | 0.005 ± 0.001 | 0.005 ± 0.001 |
| Oxalate *(971)* | *Placebo* | **0.04** | 0.24 | 0.013 ± 0.005 | 0.015 ± 0.006 | 0.014 ± 0.004 | 0.016 ± 0.007 |
|  | *Beetroot* |  |  | 0.016 ± 0.006 | 0.02 ± 0.006 | 0.016 ± 0.005 | 0.015 ± 0.004 |
| Rhamnose *(25310)* | *Placebo* | **0.01** | 0.57 | 0.001 ± 0.001 | 0.002 ± 0.001 | 0.001 ± 0.001 | 0.001 ± 0.001 |
|  | *Beetroot* |  |  | 0.001 ± 0.001 | 0.001 ± 0.001 | 0.001 ± 0.001 | 0.001 ± 0.001 |
| Threonate *(21145021)* | *Placebo* | **0.01** | 0.94 | 0.01 ± 0.004 | 0.014 ± 0.005 | 0.011 ± 0.004 | 0.011 ± 0.005 |
|  | *Beetroot* |  |  | 0.013 ± 0.005 | 0.018 ± 0.005 | 0.011 ± 0.002 | 0.013 ± 0.003 |
| Undecanoate *(8180)* | *Placebo* | **7.9×10^-4^** | 0.15 | 7.7×10^-5^ ± 5.7×10^-5^ | 1.3×10^-4^ ± 4.7×10^-5^ | 1.2×10^-4^ ± 6.2×10^-5^ | 9.1×10^-5^ ± 3.6×10^-5^ |
|  | *Beetroot* |  |  | 1.5×10^-4^ ± 4.8×10^-5^ | 1.8×10^-4^ ± 3.9×10^-5^ | 1.4×10^-4^ ± 5.5×10^-5^ | 1.4×10^-4^ ± 3. ×10^-5^ |
| Xylose *(135191)* | *Placebo* | **0.01** | **0.02** | 0.016 ± 0.008 | 0.014 ± 0.008 | 0.007 ± 0.004 | 0.011 ± 0.007 |
|  | *Beetroot* |  |  | 0.023 ± 0.013 | 0.012 ± 0.006 | 0.02 ± 0.012 | 0.017 ± 0.015 |

*Table S2: Macro-nutrient composition of the beetroot juice and placebo supplements provided to athletes [adapted from Clifford et al. (2017)].*

| **Macro-nutrient composition** | **Supplements** | |
| --- | --- | --- |
|  | **Beetroot** | **Placebo** |
|  | *(Per 250 mL serving)* | |
| Energy *(kcal)* | 81.0 | 76.8 |
| Glycaemic content *(g)* | 16.4 | 16.4 |
| Fats *(g)* | 0.4 | Trace amounts |
| Protein *(g)* | 2.8 | 2.8 |

*
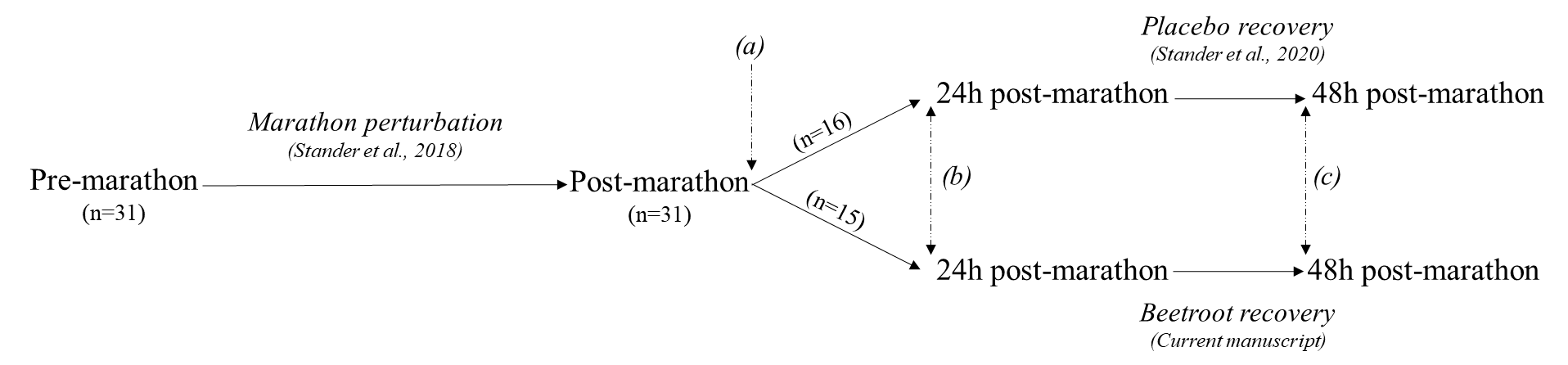
*

***Fig. S1*** *The larger scope of this investigation consists of multiple objectives:* ***Objective 1:*** *Effects of a marathon on the serum metabolome of athletes (pre- vs post-marathon; n=31) (Stander et al. 2018);* ***Objective 2:*** *Metabolic recovery without the intervention of recovery aids, by comparing pre-, post- as well as 24 h and 48 h post-marathon samples of the athletes (n=16) that ingested placebo supplements (Stander et al. 2020);* ***Objective 3:*** *Effect of beetroot juice supplementation on metabolic recovery, by comparing pre-, post- as well as 24 h and 48 h post-marathon samples of the athletes (n=15) that ingested beetroot juice supplements (current investigation);* ***Objective 4:*** *The possible added advantage of beetroot juice supplementation aided recovery versus natural (placebo) recovery, by comparing profiles of Objectives 2 and 3 (current investigation). As indicated with dashed arrows, supplements were consumed (a) on the day of the marathon (immediately after P1 sampling, ±3 h post-race, and at 20:00); (b) on the first day after the marathon (upon waking-up, with lunch, and with supper), and (c) upon waking on the second day post-marathon*


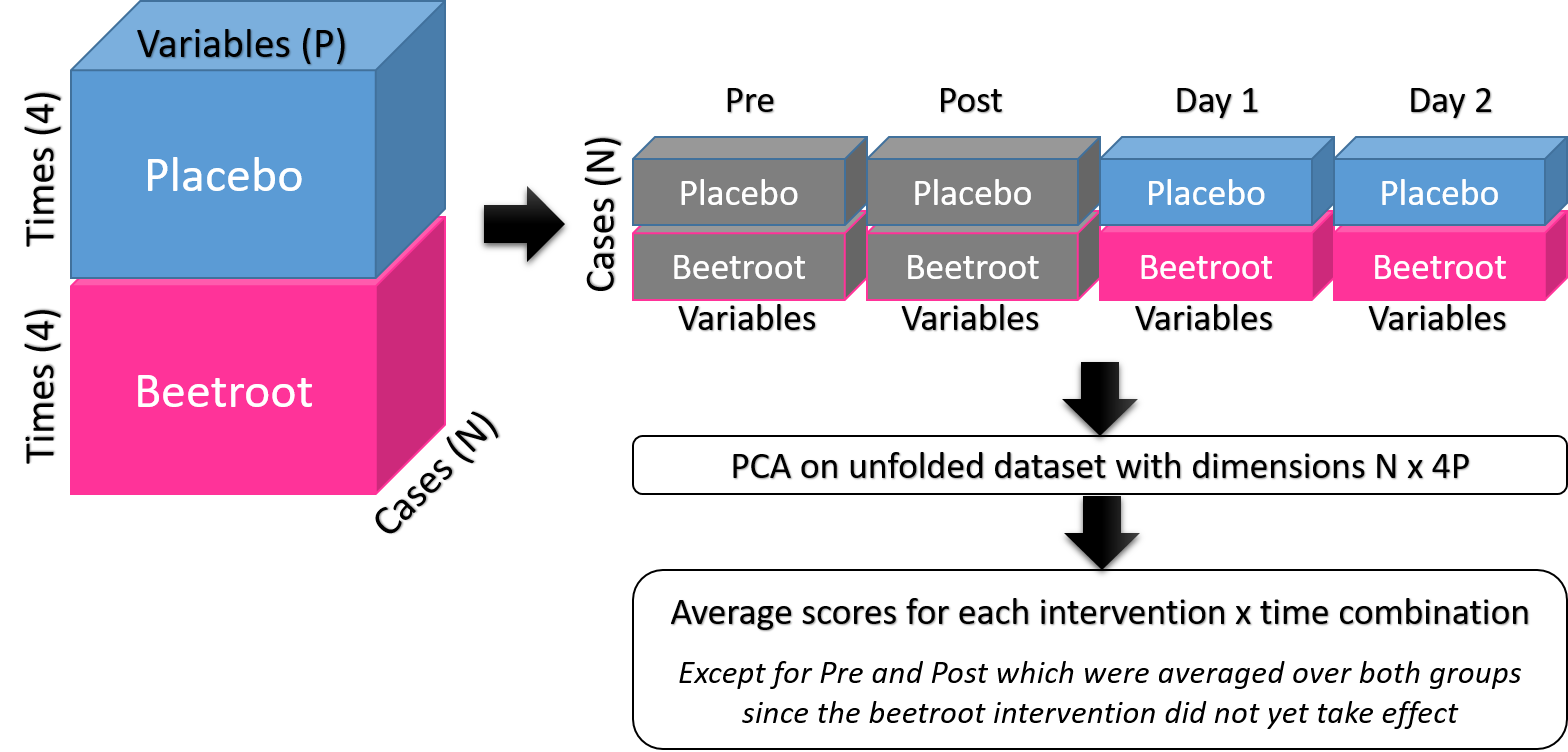
***Fig. S2:*** *A summary of the unfolded PCA of the beetroot and placebo interventions over time. Abbreviations: N: number of cases; P: number of variables.*

| 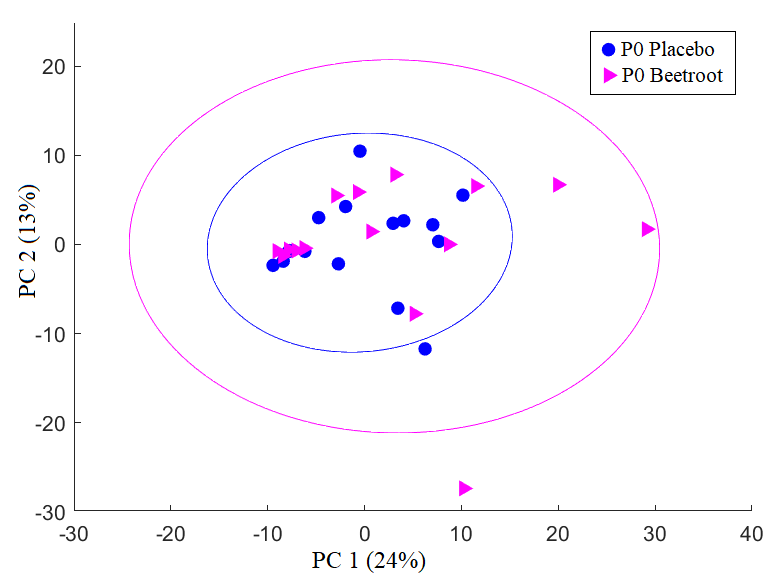(a) | 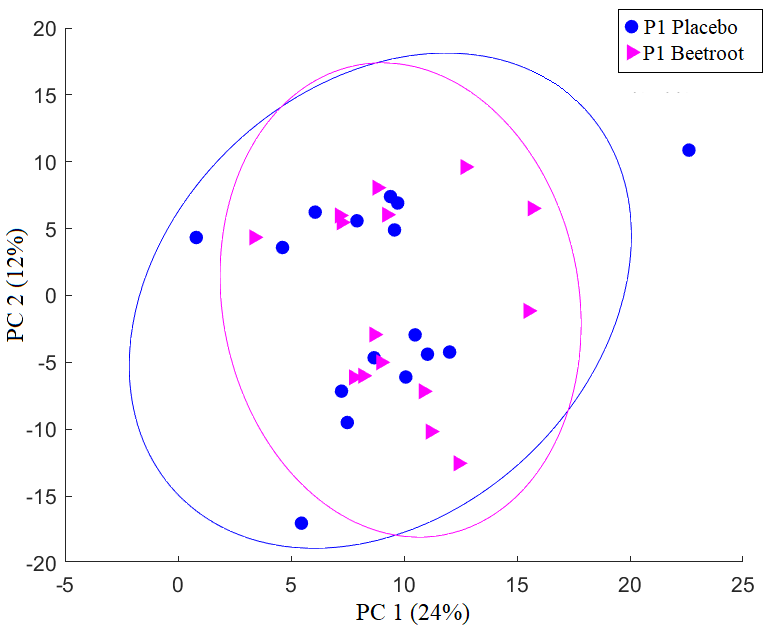(b) |
| --- | --- |
| 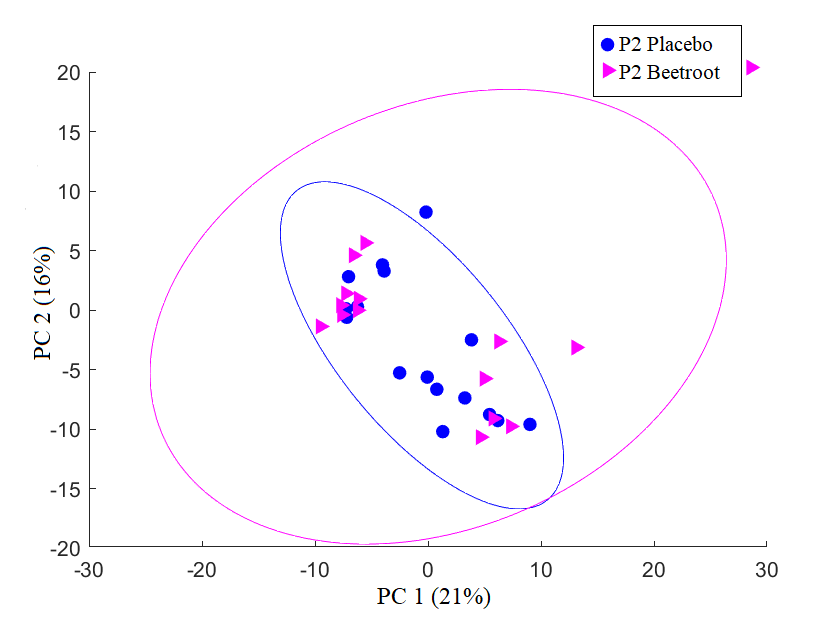(c) | 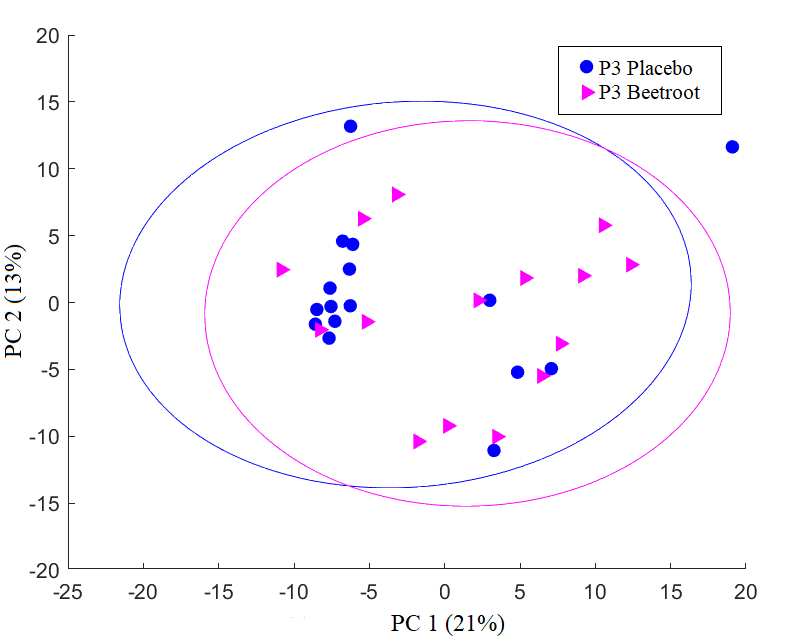(d) |

***Fig. S3:*** *Principal component analysis plots representing the inter-cohort differentiation of (a) pre-marathon [P0], (b) post-marathon [P1], (c) 24 h post-marathon [P2], and 48 h post-marathon [P3] metabolic profiles. Abbreviations: PC: principal component.*
